# 734 Use of the Burn Care Quality Platform (BCQP) for Modern Burn Care Benchmarking

**DOI:** 10.1093/jbcr/irae036.277

**Published:** 2024-04-17

**Authors:** Madeline G Lechtenberg, Jeffrey E Carter, Denise Danos, Bart D Phillips, Erica Murata, Jonathan E Schoen, Herb A Phelan

**Affiliations:** LSU Health Sciences Center - New Orleans, Lake Charles, LA; University Medical Center (LSU Health), New Orleans, LA; LSUHSC New Orleans, New Orleans, LA; BData, Inc, Edina, MN; BData, Inc., Chicago, IL; Louisiana State University Health Sciences Center- New Orleans, New Orleans, LA; LSU Health Sciences Center - New Orleans, Lake Charles, LA; University Medical Center (LSU Health), New Orleans, LA; LSUHSC New Orleans, New Orleans, LA; BData, Inc, Edina, MN; BData, Inc., Chicago, IL; Louisiana State University Health Sciences Center- New Orleans, New Orleans, LA; LSU Health Sciences Center - New Orleans, Lake Charles, LA; University Medical Center (LSU Health), New Orleans, LA; LSUHSC New Orleans, New Orleans, LA; BData, Inc, Edina, MN; BData, Inc., Chicago, IL; Louisiana State University Health Sciences Center- New Orleans, New Orleans, LA; LSU Health Sciences Center - New Orleans, Lake Charles, LA; University Medical Center (LSU Health), New Orleans, LA; LSUHSC New Orleans, New Orleans, LA; BData, Inc, Edina, MN; BData, Inc., Chicago, IL; Louisiana State University Health Sciences Center- New Orleans, New Orleans, LA; LSU Health Sciences Center - New Orleans, Lake Charles, LA; University Medical Center (LSU Health), New Orleans, LA; LSUHSC New Orleans, New Orleans, LA; BData, Inc, Edina, MN; BData, Inc., Chicago, IL; Louisiana State University Health Sciences Center- New Orleans, New Orleans, LA; LSU Health Sciences Center - New Orleans, Lake Charles, LA; University Medical Center (LSU Health), New Orleans, LA; LSUHSC New Orleans, New Orleans, LA; BData, Inc, Edina, MN; BData, Inc., Chicago, IL; Louisiana State University Health Sciences Center- New Orleans, New Orleans, LA; LSU Health Sciences Center - New Orleans, Lake Charles, LA; University Medical Center (LSU Health), New Orleans, LA; LSUHSC New Orleans, New Orleans, LA; BData, Inc, Edina, MN; BData, Inc., Chicago, IL; Louisiana State University Health Sciences Center- New Orleans, New Orleans, LA

## Abstract

**Introduction:**

In 2019, the American Burn Association (ABA) launched the Burn Care Quality Platform (BCQP), a registry tailored to create consistency across other national trauma registries. With the release of BCQP version 2 (BCQPv2) in 2021, this registry now includes data from 103 participating burn centers and 375,000 total patients as of 2021. This robust registry therefore represents a unique opportunity to measure up-to-date benchmarks for outcomes among U.S. burn centers.

**Methods:**

BCQPv2 was queried for clinically relevant burn outcomes for all data-submitting centers between the years 2018 to 2022. An additional query was run to measure outcomes for our verified, urban burn unit. Results were reported as descriptive statistics.

**Results:**

Nationally, mortality rates for all burn center admissions was 3.3%. Mean length of stay (LOS) was 3.0 days/% Total body surface area (TBSA) burned, and severe injury (defined as burn >20% TBSA) constituted 6.4% of all burn injuries nationally. Seven point nine percent of burn injuries included concomitant nonthermal traumatic injury. Payor mixes for all burn patients were 29.1% Medicaid, 15.9% Medicare, 25.6% private insurance, and 6.3% workman’s comp. National discharge dispositions followed an expected stair-step distribution when grouped by TBSA deciles. Locally, we found our mortality to be 5.1% with a mean LOS of 1.8 days/%TBSA. We saw higher proportions of severe injury (12.0%), Medicaid (41.4%), workman’s comp (10.4%), and burns with concomitant trauma (32.0%) as compared to the national findings. Additionally, higher rates of discharge home were seen on a burn-by-burn basis.

**Conclusions:**

These results represent the current state of burn care in the U.S. Locally, our burn center’s results differ in important ways, some of them potentially modifiable. Further work is being done to determine the etiologies of these differences and whether they are potentially exportable.

**Applicability of Research to Practice:**

This robust registry enables us to better evaluate the outcomes of current standards of care. This raises the possibility of improved modern burn care benchmarking and eventually better patient outcomes.

